# Interference-like patterns of static magnetic fields imprinted into polymer/nanoparticle composites

**DOI:** 10.1038/s41467-017-01861-1

**Published:** 2017-11-16

**Authors:** Zhijie Yang, Jingjing Wei, Konrad Giżynski, Myung-Geun Song, Bartosz A. Grzybowski

**Affiliations:** 1 Center for Soft and Living Matter of Korea’s Institute for Basic Science (IBS), Ulsan, 44919 South Korea; 2 Institute of Physical Chemistry, Polish Academy of Sciences Kasprzaka 44/52, 01-224 Warsaw, Poland; 3 Semiconductor R&D Center, Samsung Electronics Co., LTD., Hwaseong-si, 18448 South Korea; 4 Department of Chemistry, Ulsan National Institute of Science and Technology, Ulsan, 44919 South Korea

## Abstract

Interference of waves is important and used in many areas of science and technology but does not extend to static magnetic fields which lack the wave structure. On the other hand, magnetic fields can be spatially modulated using microstructured materials comprising magnetic and non-magnetic domains. Here, we show that when such spatial modulation is coupled to the dynamics of magnetic particles, it can give rise to interference-like patterns. These patterns are imprinted into thin polymer films by overlaying “stamps” presenting periodic arrays of magnetic and nonmagnetic regions. The structures that emerge from such a superposition are sensitive to any motions of the stamps, can depend on the history of these motions, can produce features significantly smaller than those in the stamps, and can be either planar or three-dimensional.

## Abstract (web summary)

For materials fabrication, it is most efficient to create various patterns from the same set of templates. Here, the authors show that when magnetic particles are exposed to stacks of periodic magnetic fields, they can form dynamic interference-like patterns that reflect where the magnetic fields overlap.

## Introduction

Superposition of optical, acoustic or radio waves resulting in their interference is an all-important phenomenon underlying multiple applications in spectroscopy^1^, atomic and molecular physics^2–4^, materials science^5,6^, rheology^7^, medical imaging^8^, geoscience^9^, astronomy^10^, and more. In contrast, static magnetic fields lack the wave structure and cannot be interfered—at best, these fields can be spatially modulated in multi-material systems on scales from macro-^11–14^ to microscopic^15,16^.

In this work, however, we demonstrate that when spatial modulation of static magnetic fields is coupled to the dynamics of magnetic particles, it can give rise to interference-like patterns. These patterns form in thin films of viscous polymers harboring magnetic nanoparticles and sandwiched between two periodic, magnetic “grids”. Although the emerging structures resemble optical moiré patterns^17,18^, the moirés are only optical illusions whereas the interference structures in our system are real—that is, due to field gradients created in the plane of the polymer layer, the magnetic particles are accumulating in the “crest” regions where the local fields of both grids add up, and are completely cleared from the “nadirs” where the field of only one grid is felt. In addition to static structures observed for a given arrangement of grids, this system exhibits unique dynamic properties whereby, upon grid motion, the particles can be spatially focused to various locations. Remarkably, this effect depends on the history of grid motion and has no analogy in interference optics. Overall, superposition of micropatterned magnetic fields coupled to the guided assembly of magnetic particles can broaden the currently available repertoire^15,16,19–25^ of methods for the assembly and/or manipulation of magnetic colloids or nanoparticles, and open new avenues for the fabrication of magnetic/nonmagnetic composites.

## Results

### Fabrication and system set-up

**Fig. 1:**
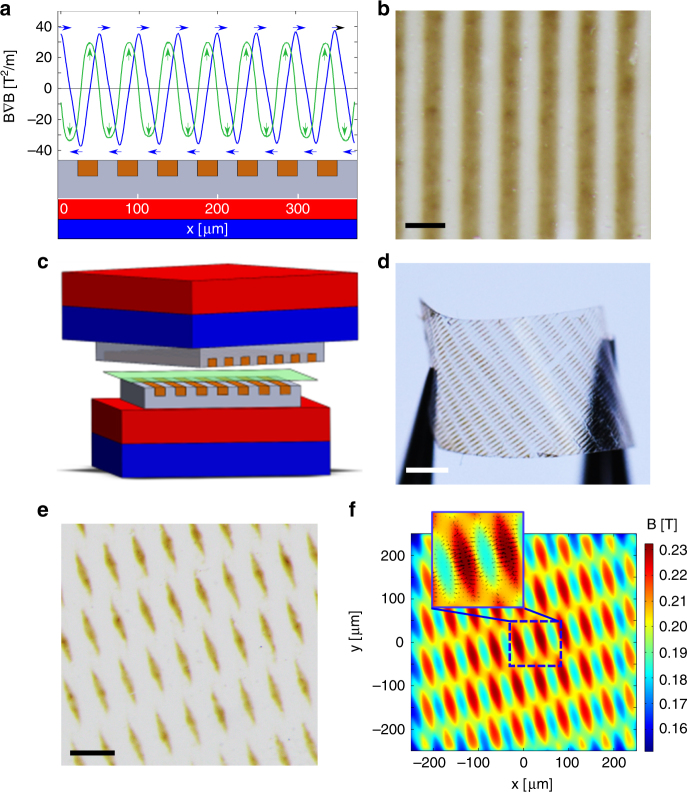
Directed assembly of magnetic nanoparticles by micropatterned magnetophoretic forces. **a** Profile of horizontal (blue line) and vertical (green line) components of magnetic force above a magnetic (gray)/nonmagnetic (maroon) composite stamp placed on a NdFeB permanent magnet. Arrows indicate the direction of the resulting forces (towards the magnetic regions). The magnetic features used in the experiments were typically on the order of 100 μm. **b** A PMMA film containing 12 nm Fe_3_O_4_ magnetic nanoparticles assembled over an array of magnetic lines in one PDMS stamp. Scale bar = 200 μm. **c** Scheme of experimental setup for interfering the fields produced by two magnetically patterned PDMS stamps placed between two permanent magnets. One of the stamp-magnet pairs is stationary and the other is manipulated by a motorized translational and/or rotational stage. **d** A freestanding PMMA film with a pattern of Fe_3_O_4_ NPs produced by two arrays of parallel lines inclined by *θ* = 30^o^ with respect to one another. **e** Top view of a pattern of Fe_3_O_4_ NPs forming between two arrays of lines placed at an angle *θ* = 30^o^ with respect to one another. Note the particles are localizing exclusively to the rhomboidal regions at lines’ intersections. **f** The corresponding magnetic field distribution in the plane of the film. Horizontal *x, y*-components of gradient force over high- and low-field regions are marked with black arrows in the inset—these forces drive the particles to the regions of lines’ intersections. Scale bars in **b**,** d**, and **e**, are 200 μm, 1 mm and 500 μm, respectively

The experimental setup is illustrated in Fig. 1 (also see Supplementary Fig. 1). First, magnetic stamps several centimeters in size and with a desired micropattern embossed in bas-relief were fabricated by conventional soft-lithography protocols^26^ using poly(dimethylsiloxane), PDMS, doped with 33 wt% of iron microparticles (5–9 μm, Sigma-Aldrich). The spaces between the stamp’s features were then filled with PDMS containing 10 wt% of diamagnetic graphite microparticles (7–11 μm, Alfa Aesar). The composite structure thus prepared had both non-magnetic and magnetic regions near its surface. When this magnetic/nonmagnetic “grid” was placed on top of a 1.32 T permanent magnet (NdFeB block, K&J Magnetics SBCX86-IN, magnetized along the short dimension), the field was weakened over the graphite-filled regions and concentrated over iron-containing domains—this field profile translated, in turn, into magnetic forces directed towards the magnetic regions (Fig. 1a, b; also see results of simulations in the Supplementary Note 1). Next, two magnetic/non-magnetic PDMS slabs made in this way were placed face-to-face to modulate an otherwise uniform field between two permanent magnets (Fig. 1c). The 100–200 μm gap between the PDMS surfaces was filled with a toluene solution containing 5 wt% of poly(methyl methacrylate) and 0.75 wt% of Fe_3_O_4_ nanoparticles (12 nm in size, 7% polydispersity, Supplementary Fig. 2), and the interference patterns were produced by adjusting the mutual positions of the magnetic-/non-magnetic grids. Once formed, the patterns were solidified into freestanding films (Fig. 1d) by solvent evaporation. For more experimental details, see Supplementary Methods.

### Micropatterned magnetophoretic forces

Figure 1e shows a nanoparticle pattern formed in a PMMA film between two sets of parallel magnetic lines oriented at *θ* = 30^o^ angle with respect to one another. As seen, the magnetic particles—initially, uniformly distributed—localize exclusively to the rhomboidal regions at the lines’ intersections. At first sight, it might appear counterintuitive why the NPs are cleared not only from over the diamagnetic regions of the stamps but also from those corresponding to the non-overlapping portions of the magnetic lines—in fact, the magnetic flux density, ${\bf B}$, is clearly non-zero over all of these regions (Fig. 1f, see also Supplementary Figs. 3 and 4). However, what dictates particle’s motions is not **B** itself but magnetic forces proportional to the field gradient in the plane of the film, ${\mathbf{F}}_{\mathbf{m}} \propto {\mathrm{\nabla }}{\mathbf{B}}$. As shown in the inset to Fig. 1f, the ${\mathbf{F}}_{\mathbf{m}}$ vectors are directed towards the rhomboidal line intersections. Based on this phenomenon, an analogy to the way optics may be formulated as follows–if periodic structures in the PDMS stamps are “waves” with “crests” corresponding to the magnetic regions, their “interference” is “constructive” where the crests overlap and is “destructive” otherwise.

### Formation of moiré-like patterns

**Fig. 2:**
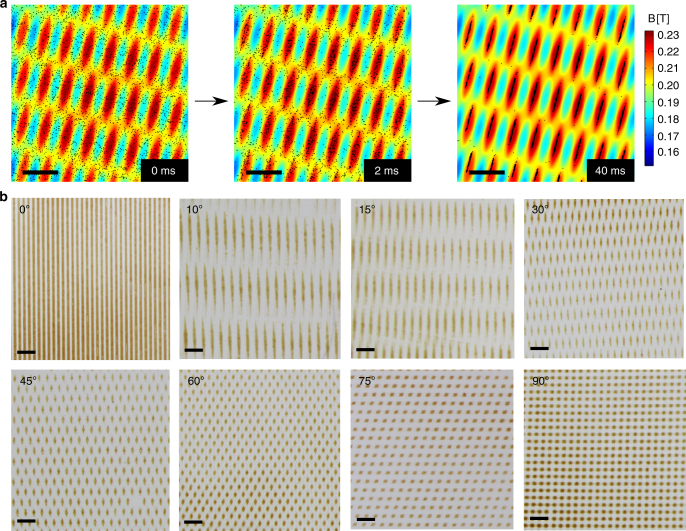
Moiré-like patterns forming by the superposition of static magnetic fields. **a** MD simulation of magnetic NPs moving into high-field regions produced by two arrays of parallel magnetic lines placed at *θ* = 30^o^ with respect to one another. See also Supplementary Movies 2 and 5. **b** Optical micrographs of nanoparticle/polymer films forming between two line patterns at different angles or rotation (from *θ* = 0^o^ to *θ* = 90^o^). Scale bars in **a** and **b** are 100 μm and 500 μm, respectively

**Fig. 3:**
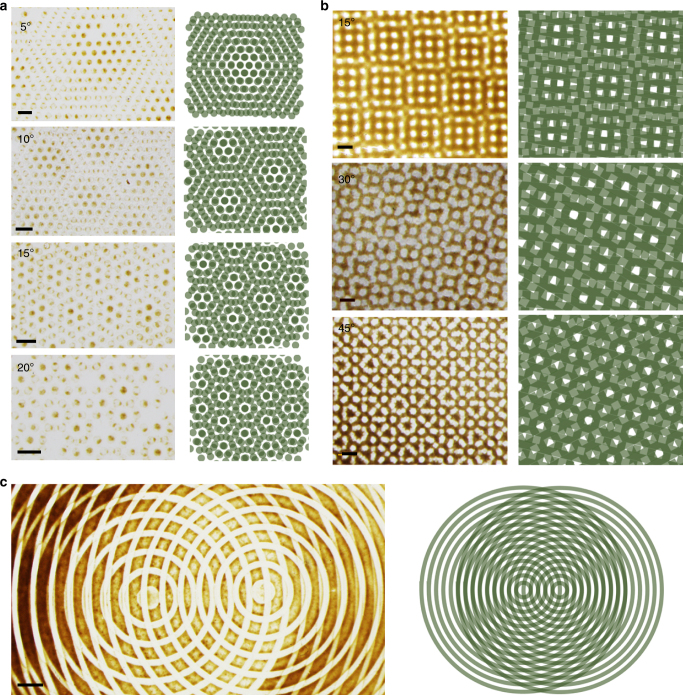
Complex patterns from superposition of simple individual fields. The structures shown were prepared by overlapping **a**, Two hexagonal arrays of circular dots at angles 5°, 10°, 15°, and 20°. **b** Two arrays of squares at angles 15°, 30° and 45°. **c** A circular structure formed between two arrays of concentric circles. The green miniatures are moiré patterns which are similar—but not identical—to the magnetic structures we observe. In the experimental images in **a**, note that some of the features are much smaller/thinner than the original dots. All scale bars = 500 μm

Figures 2 and 3 demonstrate that the NP patterns observed for different types and/or different orientations of the PDMS stamps are similar to the well-known moiré patterns. It should be stressed, however, that the similarity is only visual since in the moirés, the non-overlapping regions of individual patterns do not become transparent (unlike in our magnetic structures, in which these regions are cleared of any NPs, see above). We also note that the sizes of the smallest features that are resolved in the emerging, magnetic-NP patterns can be smaller than the sizes of features in the superimposed PDMS stamps–this is so because the particles are attracted to the regions of overlap between the “bottom” and “top” patterns, and for some mutual pattern orientations, these loci of overlap can be very small/thin.

### “Focusing” of magnetic particles

**Fig. 4:**
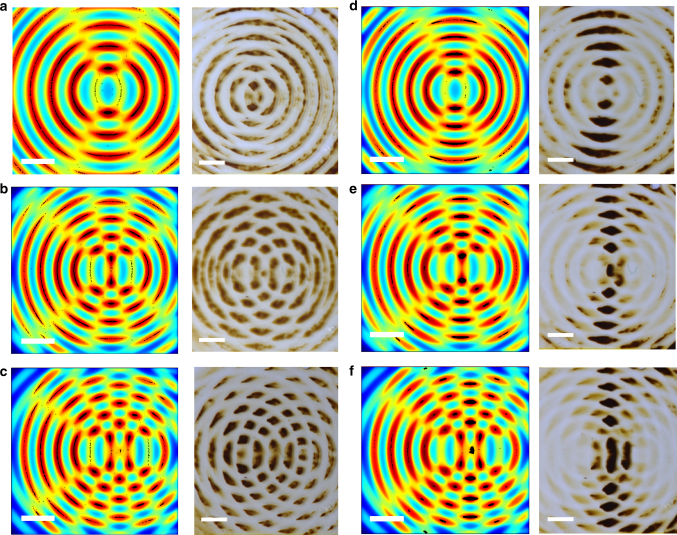
“Focusing” of magnetic particles upon motion of the field-templating grids. The patterns form between two stamps, each presenting an array of concentric magnetic circles. In **a**–**c** the circle arrays are immediately placed in the orientations shown. In **d**–**f**, they are first put on top of one another, and then one stamp is slowly moved sideways. Experimental images are accompanied by the results of MD simulations showing field intensity and distribution of particles (black spots in field maxima). Also see Supplementary Movie 4 visualizing the motions of particles in MD simulations. Scale bars in simulation and experimental images are 100 μm and 2 mm, respectively

Another fundamental difference with the moirés is that the nanoparticle structures in our systems depend not only on the orientation of the guiding PDMS patterns, but also on how these patterns were brought into a particular position. This “history dependence” is illustrated in Fig. 4 which compares patterns formed under the influence of the same two grids of magnetic circles but immediately placed at center-to-center distances *d* = 2, 4 or 6 mm vs. patterns initially placed on top of one another and only then slowly (ca. 2 µm/s) displaced to the same distances. The distributions of nanoparticles that emerge in these two cases are markedly different—notably, for the dynamic pattern, the particles are not present under all intersections of circles, but are “focused” to the intersections lying along the line bisecting and perpendicular to the line joining the centers of the two circle arrays. We make three additional observations about these experiments. First, the focusing becomes more pronounced as the circles are moved further apart. Second, the particles are moved more efficiently by the moving grids if their sizes are larger and their magnetic susceptibilities higher–to this end, focusing experiments were typically performed with Fe_3_O_4_ “supraparticles”^27^ ca. 300 nm in diameter (cf. Supplementary Methods). Third, similar effects are observed for other types of patterns (cf. Supplementary Fig. 5 for two arrays of magnetic lines being rotated).

## Discussion

To understand the formation of the observed patterns–and especially, the history dependence—in quantitative detail, we considered a theoretical model in which instantaneous field distributions and field gradients at a particular orientation of the grids were modeled by the finite-element method (in the COMSOL software package^28^) and the motions of the nanoparticles in response to these distributions were calculated by Molecular Dynamics (MD). In the MD simulations, each nanoparticle *i* of mass $m_i$ moved according to Newtonian equations of motion, $m_i{\bf{\ddot x}}_{\mathrm{i}} = {\mathbf{F}}_{{\mathrm{drag}}}^{\mathrm{i}} + {\mathbf{F}}_{\mathrm{m}}^{\mathrm{i}} + {\mathbf{F}}_{{\mathrm{dd}}}^{\mathrm{i}} + {\mathbf{F}}_{{\mathrm{rep}}}^{\mathrm{i}}$. In this expression, the drag force in a viscous medium is given by the Stokes’ formula, ${\mathbf{F}}_{{\mathrm{drag}}}^{\mathrm{i}} = 6\pi \nu R_pv_{\mathrm{p}}$, where $\nu$ is viscosity of the polymer’s toluene solution (taken as 0.001 Pa s), $R_p$~0.5 μm is the approximate size of the magnetic supraparticles, and **v**_p_ is particle’s velocity. The magnetic force imparted on the particle by the fields of the magnetic grids is ${\mathbf{F}}_{\mathrm{m}}^{\mathrm{i}} = {\mathbf{m}}_{{\mathrm{eff}}}^{\mathrm{i}} \cdot \nabla {\mathbf{B}}$. In this equation, **B** stands for the magnetic flux density, and the particle’s “effective” magnetic moment^29^ is ${\mathbf{m}}_{{\mathrm{eff}}}^{\mathrm{i}} = (\chi _{\mathrm{p}}V_{\mathrm{p}}/\mu _f){\mathbf{B}}$, where $V_{\mathrm{p}}$ is particle volume, the permeability of the PMMA polymer is approximated as close to that of free space, $\mu _f \approx \mu _0$ (assumption valid for low concentration of magnetic particles), and magnetic susceptibility of the particle is calculated as in ref. ^30^, $\chi _{\mathrm{p}} = \frac{{M_s\mu _0}}{{|\mathbf{B}|}}$ if $\frac{{|\mathbf{B}|}}{{\mu _0}} \ge \frac{{M_s}}{3}$ and $\chi _{\mathrm{p}} =$3 otherwise (with saturation magnetization of iron oxide, *M*_s_ = 4.78×10^5^ A/m). The other magnetic force term, ${\mathbf{F}}_{{\mathrm{dd}}}^{\mathrm{i}}$, accounts for the interactions of a particle’s induced moment with the induced moments of all other particles present in the system:

$$\begin{array}{*{20}{l}}{\mathbf{F}}_{{\mathrm{dd}}}^{\mathrm{i}} = \mathop{\sum}\limits_{\scriptstyle {\mathrm{j}} = 0\hfill \atop \scriptstyle {\mathrm{j}} \ne {\mathrm{i}}}^{N} {\frac{{3\mu _0}}{{4\pi \left| {{\mathbf{r}}_{{\mathrm{ij}}}} \right|^4}}} \left[\left( {\hat {\mathbf{r}}} \times {\mathbf{m}}_{{\mathrm{i}},{\mathrm{eff}}} \right)\times {\mathbf{m}}_{{\mathrm{j}},{\mathrm{eff}}} + \left({\hat {\mathbf{r}}} \times {\mathbf{m}}_{{\mathrm{i}},{\mathrm{eff}}} \right)\right.\\ \left.\times {\mathbf{m}}_{{\mathrm{i}},{\mathrm{eff}}} - 2{\hat {\mathbf{r}}}\left({{\mathbf{m}}_{{\mathrm{i}},{\mathrm{eff}}} \times {\mathbf{m}}_{{\mathrm{i}},{\mathrm{eff}}}}\right) + 5{\hat {\mathbf{r}}}\left({ \left( {{\hat {\mathbf{r}}} \times {\mathbf{m}}_{{\mathrm{i}},{\mathrm{eff}}}} \right) \cdot \left( {\hat {\mathbf{r}}} \times {\mathbf{m}}_{{\mathrm{i}},{\mathrm{eff}}} \right)}\right) \right],\end{array}$$

where $\hat {\mathbf{r}}$ is a unit vector pointing from the i-th to the j-th particle and $\left| {{\mathbf{r}}_{{\mathrm{ij}}}} \right|$ denotes the distance between their centers. To prevent particle overlap, a soft repulsive force term is included, $${\mathbf{F}}_{{\mathrm{rep}}}^{\mathrm{i}} = - \mathop {\sum}\nolimits_{\scriptstyle {\mathrm{j}} = 0\hfill \atop\\ \scriptstyle {\mathrm{j}} \ne {\mathrm{i}}\hfill }^N {F_0 \cdot {\mathrm{exp}}} \left( { - \kappa \frac{{\left| {{\mathbf{r}}_{{\mathrm{ij}}}} \right| - 2R_p}}{{2R_p}}} \right)\hat {\mathbf{r}}$$, where *F*_0_ = 1×10^−8^ and *κ* = 100 are parameters adjusted to compensate the particle-particle interactions in high field regions. Gravity and Brownian effects are not accounted for since they are small compared to the magnetic interactions (see Supplementary Movie 1). For *N* particles, we then solved a system of second-order, coupled ordinary differential equations (ODE) by decomposing each into a subsystem of two first-order ODEs and integrating using the Velocity–Verlet algorithm^31^.

The results of the simulations are summarized in Figs. 1 and 2 for static patterns and in Fig. 4 for dynamic ones (also see Supplementary Fig. 5 and Supplementary Movies 2–8). As already noted, the magnetically patterned stamps establish field gradients in the plane of the film and the magnetic particles move towards and localize at the nearby maxima. Additionally, when the patterns move with respect to one another, the locations and spatial extents of the maxima change dynamically—in particular, the maxima become smaller—these effects are vividly illustrated in Supplementary Movies 3, 4 and ultimately lead to the “focusing” of particles over shrinking high-field regions. Interestingly, when the patterns are moved too rapidly, the particles immersed in the viscous medium do not have enough time to respond to/follow the changes in the magnetic field landscape and are left behind in the local maxima (see Supplementary Movie 5).

**Fig. 5:**
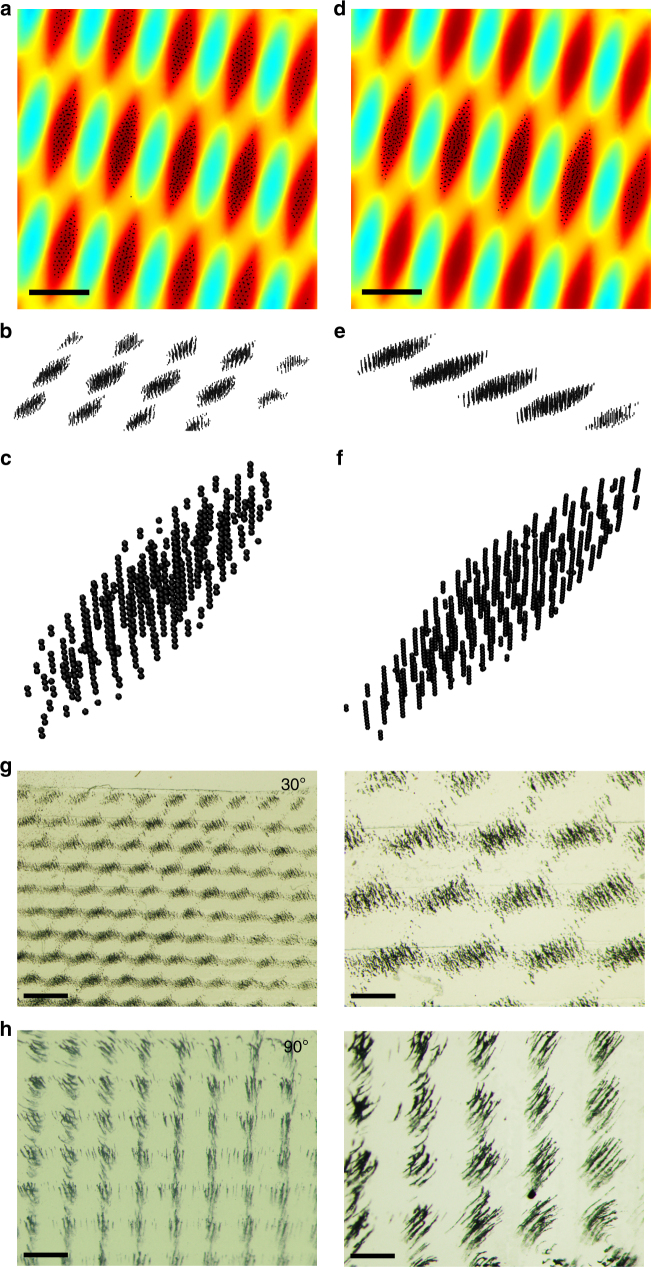
3D moiré-like magnetic patterns in simulations and in experiment. The simulations were performed for two stamps of parallel line features and **a**–**c** “statically” placed at *θ* = 30^o^ on top of one another, and **d**–**f** “dynamically” rotated (in discrete steps of 2^o^ per 2 ms) from *θ* = 0 ^o^ to 30^o^. In both cases, the particles form discrete chains, as emphasized by perspective/inclined views in **b**, **c**, **e**,** f**. Number of particles in each simulation was 5000. Scale bars = 50 µm. See also Supplementary Movies 5 and 6. **g**, **h** Optical micrographs of patterns in which Fe microparticles form discrete vertical chains over the regions of field maxima. Two patterns of 200-μm-wide, parallel magnetic lines were placed at angles **g**, *θ* = 30^o^ and **h**, *θ* = 90^o^. Scale bars are 500 μm for the images in the left column and 200 μm for the images in the right column

Finally, we comment on the role of dipole-dipole interactions which, in the model described above, are captured by the ${\mathbf{F}}_{{\mathrm{dd}}}^{\mathrm{i}}$ term. MD simulations extended to three dimensions (Fig. 5a–f, Supplementary Figs. 6–8 and Supplementary Movies 5, 6) confirm^32^ that such interactions are expected to give rise to the chaining of the particles perpendicular to the plane of the field-templating stamps, and to repulsive interactions causing these chains to form “open-lattice” arrays within the field-maxima islands of our interference-like patterns. For a given strength of the magnetic field, the spacing between these chains is expected to decrease with increasing confinement (i.e., with decreasing island size) while the overall quality of the patterns is expected to be better in less viscous media (so that the drag forces do not prevent the particles from entering the field maxima) and, in dynamic patterns, for lower rotation rates (see Supplementary Note 2). To realize such secondary chain arrays within moiré-like patterns in experiments, we used carbonyl-iron microparticles (5–9 μm from Sigma-Aldrich, magnetic susceptibility^33^ 4.5) for which magnetic moments were higher and the dipole-dipole forces stronger than for Fe_3_O_4_ nanoparticles or nanoparticle supraspheres. The structures obtained by superposition of two patterns of magnetic lines at angles *θ* = 30^o^ (Fig. 5g) and *θ* = 90^o^ (Fig. 5h) clearly show distinct magnetic “whiskers” over the local field maxima.

In summary, we have described a system in which local modulation of static magnetic fields couples with the dynamics of magnetic nanoparticles to produce interference-like patterns, sometimes ones that would be hard to predict based on the geometry of the field-templating stamps (cf. a rotor example in Supplementary Fig. 9). Aside from fundamental interest, we suggest this approach can be used for the fabrication of magnetic-nonmagnetic composites, whereby markedly different periodic structures could be obtained from the same imprinting patterns placed at slightly different mutual orientations. Another potential extension is the use of “focusing” phenomenon in conjunction with various magnetic trapping modalities^34^. In these and other applications, one of the challenges will be to miniaturize the system—our calculations indicate that Brownian effects in a viscous polymer medium are small compared to magnetic forces and sub-micron patterns (perhaps below the diffraction limit of light) could be resolved provided the spacing between the PDMS stamps is commensurate with the very small sizes of magnetic features (Supplementary Note 1).

### Data availability

Data and computer codes that support the findings of this study are available from the corresponding author upon request.

## Electronic supplementary material

Supplementary Information

Description of Additional Supplementary Information

Supplementary Movie 1

Supplementary Movie 2

Supplementary Movie 3

Supplementary Movie 4

Supplementary Movie 5

Supplementary Movie 6

Supplementary Movie 7

Supplementary Movie 8
