# Supplementary material for: Interference-like patterns of static magnetic fields imprinted into polymer/nanoparticle composites
Source: Nat Commun. 2017 Nov 16;8:1564. doi: 10.1038/s41467-017-01861-1 (PMC5691180; doi:10.1038/s41467-017-01861-1)
Supplement: Supplementary file 1 — Supplementary Information [file 41467_2017_1861_MOESM1_ESM.pdf]

SUPPLEMENTARY INFORMATION

Supplementary Methods.

Synthesis of magnetic Fe_3O_4 nanoparticles. Monodisperse iron oxide NPs were synthesized by modifying the method reported by Hyeon *et al.*[1]. First, iron oleate precursors were prepared as follows: 10.8 g of iron(III) chloride hydrate, 36.5 g of sodium oleate, 40 mL of deionized (DI) water, 40 mL of ethanol, and 80 mL of hexane were mixed in a 500 mL three-neck flask. The mixture was refluxed at 60 ^{o}C for 4 h and the iron oleate (of dark red-black appearance) was obtained in the upper organic phase. The hexane solution containing iron oleate was further washed three times with warm DI water (~50 ^{o}C) and separated in a separatory funnel. The viscous product was obtained by evaporating hexane in a rotary evaporator. A stock precursor solution with a concentration of 0.5 mol kg^{-1} was prepared by adding 1.5 g of octadecene to each gram of iron oleate. In a typical synthesis of 12 nm Fe_3O_4 nanocrystals, 4.8 g of precursor solution was mixed with 0.76 g oleic acid and with 6.0 g octadecene. The mixture was heated to 110 ^{o}C and maintained at this temperature for 60 min under N_2 flow. It was then heated to the boiling point (~315 ^{o}C) and was kept at this temperature for 30 min. After letting it cool down to room temperature, the suspension of NPs was washed five times using acetone/hexane (1:1 *v/v*) by sedimenting (using centrifugation at 7800 r.p.m. for 5 min) and redispersing. Finally, the Fe_3O_4 NPs were weighted and redispersed in toluene with nanocrystal concentration of 60 mg mL^{-1}.

Synthesis of magnetic Fe_3O_4 supraparticles. In a typical experiment, 200 μL of chloroform containing 5 mg of 12-nm Fe_3O_4 nanoparticles was added to an aqueous solution containing 18 mg of dodecyl trimethyl ammonium bromide (DTAB). The oil/water mixture was emulsified by vortex agitation (2000 r.p.m.) for 30 s. Subsequently, 5 mL of ethylene glycol (EG) solution containing 0.4 g polyvinylpyrrolidone (PVP, *MW* = 40000) was added rapidly to the emulsion and

subjected to vortex agitation for another 30 s. The emulsion was then heated to 70 ^{o}C under N_2 protection and kept at this temperature for 15 min to evaporate the oil phase, resulting in a water/EG mixture. The suspension was then allowed to cool down to room temperature. The resulting nanoparticle assemblies were washed twice with ethanol and redispersed in water. The sizes of these "superparticles" were ca. 300 nm as determined by TEM and dynamic light scattering.

Fabrication of the composite PDMS stamp. PDMS (Sylgard 184 Silicone Elastomer, Dow Corning) pre-polymer and crosslinker (curing agent) were mixed in a 10:1 *w/w* ratio. For the preparation of iron-filled PDMS, iron microparticles (5-9 μm, Sigma-Aldrich) were mixed with the pre-polymer at a weight ratio of 1:3. For the preparation of graphite-filled PDMS, graphite microparticles (7-11 μm, Alfa Aesar) were mixed with the pre-polymer at a weight ratio of 1:10. Both mixtures were degassed in a vacuum chamber for 60 min to remove any air bubbles. The iron-filled PDMS was then gently poured onto a four-inch silicon wafer supporting photolithographically patterned SU8-2100 photo resist (MicroChem), followed by degassing under vacuum for another 30 min. After curing at 65 ^{o}C over 12 h, the solidified, iron-filled PDMS stamp was gently peeled off the master. The grooves between the stamp's features were filled with graphite/PDMS composite. The PDMS/graphite mixture was pressed against a glass slide and cured, resulting in an approximately flat surface of the stamp with periodic magnetic/nonmagnetic regions.

Assembly of patterns of chained-up iron microparticles. To form patterns such as those shown in **Figures 5g,h** in the main text, two magnetic/nonmagnetic PDMS stamps (each patterned with an array of 200-μm-wide, parallel magnetic lines spaced by 200 μm) were covered with a ~ 25 μm adhesive tape (3M). One of the stamps was placed pattern-up on top of a 1.32 T permanent magnet

(NdFeB block, K&J Magnetics SBCX86-IN, magnetized along the short dimension) and the other was attached (pattern down) to a translational/rotational stage (Thorlabs). The angle between the two arrays of lines was adjusted to a desired value and the gap between the stamps' surfaces was initially 1 mm. Then, 100 μL of poly (dimethyl siloxane), PDMS, pre-polymer and crosslinker (10:1 *w/w*) containing 1% *w/w* of iron microparticles (carbonyl-iron powder, 5-9 μm, Sigma-Aldrich) was injected into this gap and the upper pattern was slowly lowered until the gap was 100-200 μm. The particles were allowed to organize in response to the imposed fields for 1 min and the PDMS was subsequently thermally cured (using 150 ^{o}C heatgun for ca. 30 min).

Characterization techniques. TEM characterization of nanoparticles was performed on a JEOL JEM 2100 microscope operating at 200 kV. Samples were prepared using 400-mesh, carbon-coated copper grids (Ted Pella Inc.). The polymer/nanoparticle composites films were imaged on a standard Olympus microscope.

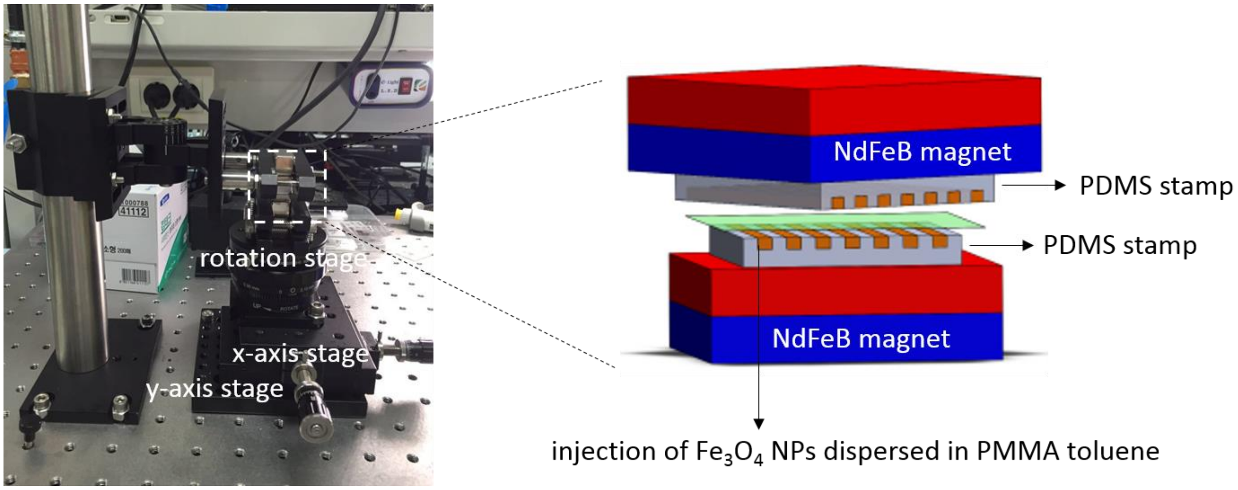

Supplementary Figure 1. Experimental setup used in experiments.

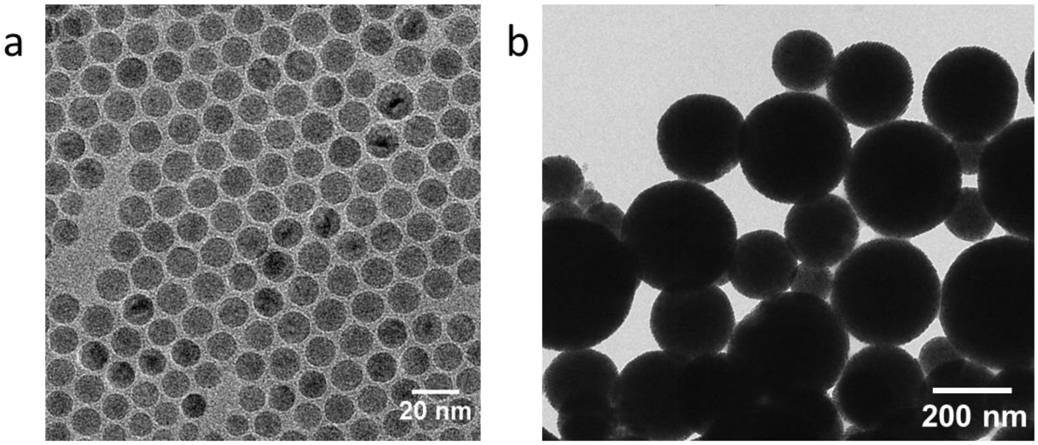

Supplementary Figure 2. Representative TEM images of magnetic Fe_3O_4 nanoparticles (ca. 12 nm in diameter) and Fe_3O_4 supraparticles (ca. 300 nm in diameter) used in experiments.

Supplementary Note 1: Additional theoretical considerations in two dimensions.

As narrated in the main text, spatiotemporal distribution of iron oxide nanoparticles was analyzed using a Molecular Dynamics (MD) model. To recreate patterns observed in experiments we typically used two dimensional version of the model as it accelerated computations yet still allowed to observe spatial organization of particles into patterns both in static and dynamic arrangements as shown in Figure 2a and 4 in the main text.

Furthermore, using FEM methods, we investigated the role of magnetic forces acting in the direction perpendicular to the plane along which interference-like patterns are observed. We compared vertical and horizontal flux density and gradient force profiles at different distances from a surface of a single stamp (Supplementary Figure 3) or pair of stamps (Supplementary Figure 4). Based on these calculations we considered the factors that need to be taken into account when miniaturizing our interference patterns. One observation based on Supplementary Figure 3 and 4 is that the field profiles become more uniform and the magnetic forces less effective as the distance from the patterned stamp increases – this mean that for smaller features, the spacing between the stamps should be decreased to still observe well-resolved interference patterns.

In another scaling argument, we considered the critical size of the particles, D_p, below which Brownian effects would dominate magnetic gradient forces. This size can be estimated based on a criterion $|\mathbf{F}_m| D_p \leq kT$.[2] We found that for 1μm features spaced at 1 μm, D_p = 25nm, implying that our method is limited predominantly by the size of the features and the thickness of nanoparticle solution layer.

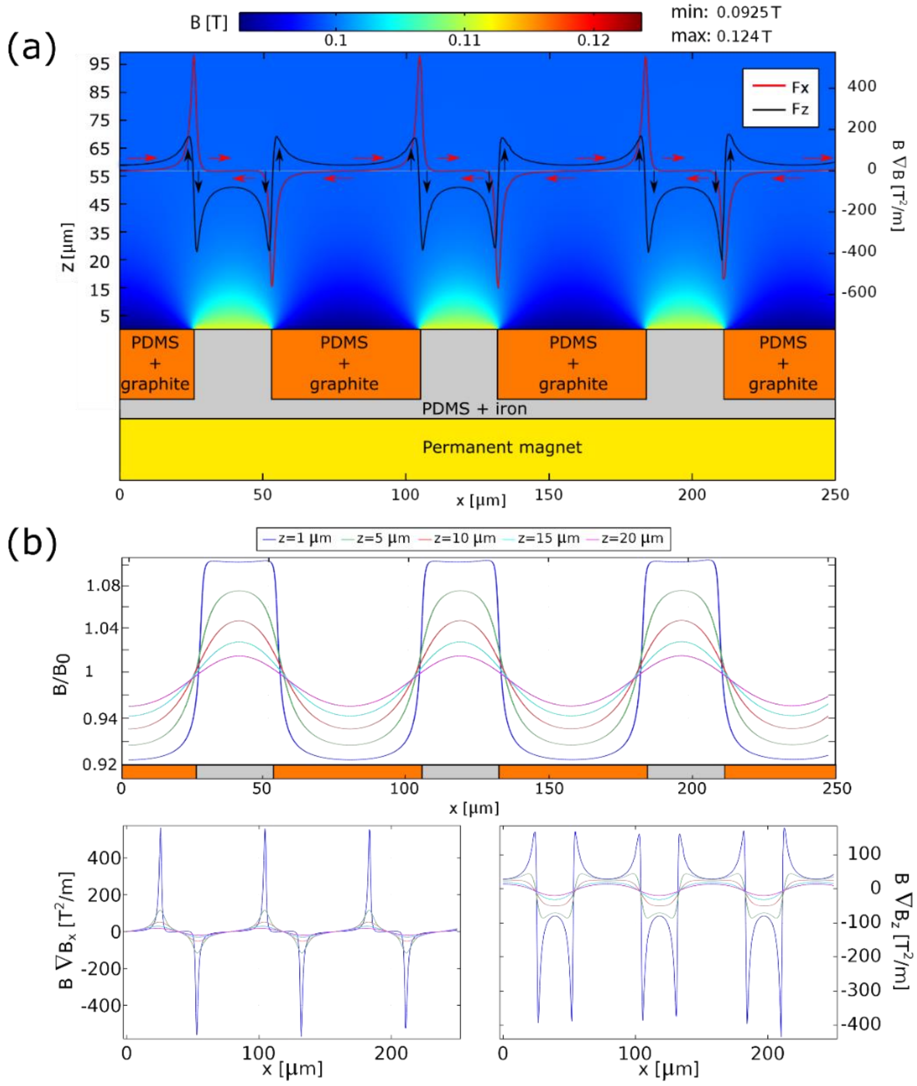

Supplementary Figure 3. Force products at different heights above magnetic pattern. a, Magnetic field distribution at the surface of the composite PDMS stamp placed on a large permanent magnet producing a nearly uniform field of 0.1 T in the area of interest. Red and black lines correspond to, respectively, the horizontal and the vertical components of the force product calculated at $z = 1$ μm. Arrows indicate direction of the forces. **b,** Magnetic flux density normalized to the flux at the surface of permanent magnet $\mathbf{B}_0$ (top), horizontal (bottom left) and vertical (bottom right) components of force product at five distances from the patterned surface.

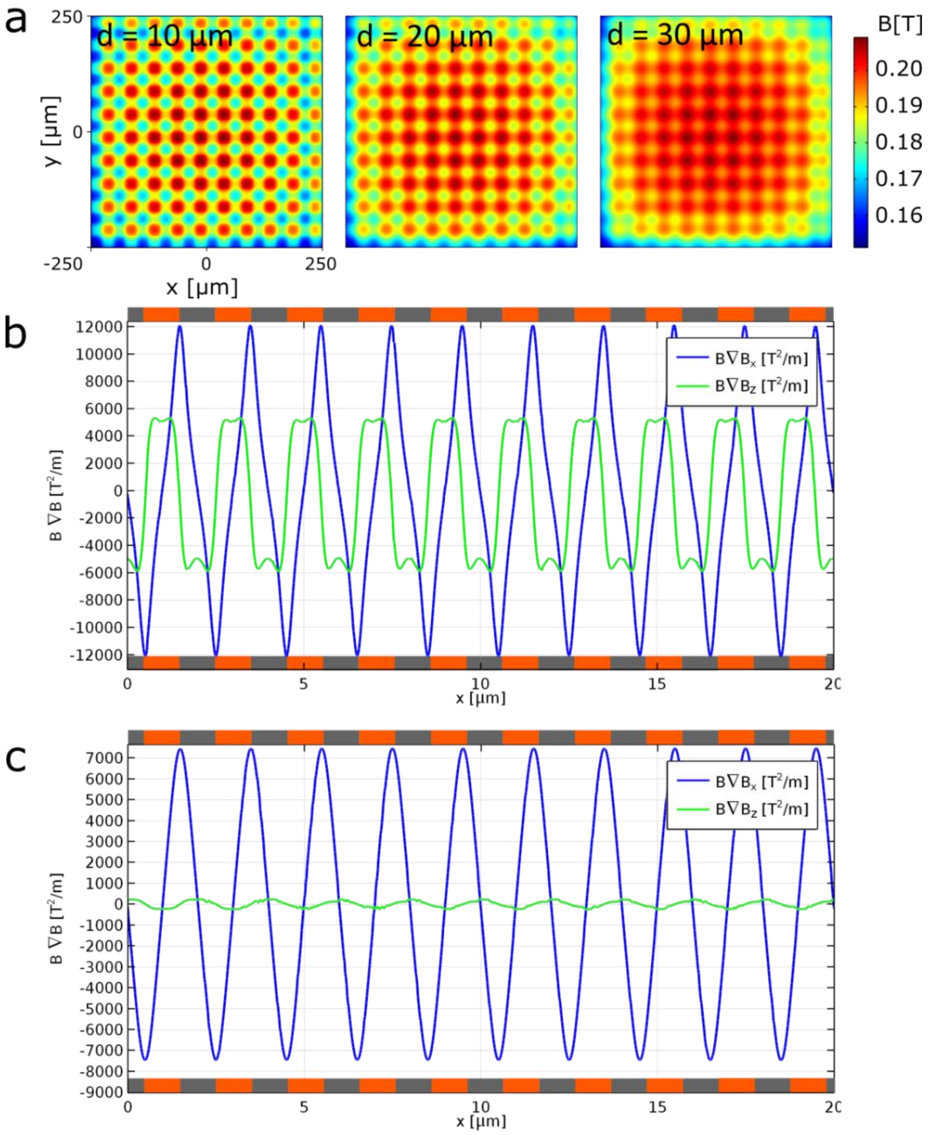

Supplementary Figure 4. Magnetic flux density and force products between two PDMS stamps. a, Interference of magnetic field modulated by two PDMS stamps with 25 µm line features. The stamps are rotated by 90° with respect to each other and spaced by 10 µm, 20 µm and 30 µm. **b,c** Force products in *x* and *z* directions between two stamps with 1 µm line features, measured at distance of **b,** 0.25 µm and **c,** 0.5 µm from the surface of the bottom stamp. The total spacing between stamps is 1 µm.

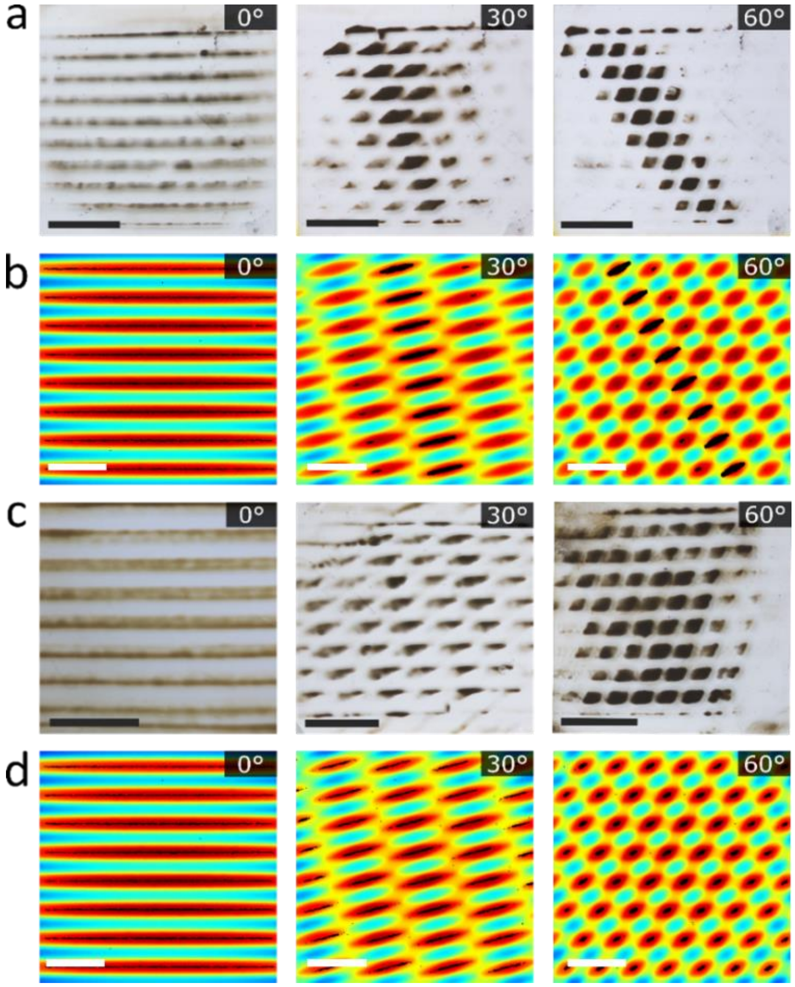

Supplementary Figure 5. An additional example of "focusing" of magnetic particles upon motion of the field-templating grids. Spatial distribution of magnetic particles between two stamps – each presenting an array of parallel magnetic lines – with rotation angle changed dynamically from 0° to 60° observed in experiments **a,** and simulations **b.** The corresponding structures obtained by static patterning are shown in **c** and **d**. Scale bars in experimental and theoretical pictures are 5 mm and 100 µm, respectively. Number of particles used in each simulation is 4000.

Supplementary Note 2: Additional theoretical considerations in three dimensions.

Next, we consider the importance of interactions between the dipoles induced in the magnetic particles. The first question we are interested here is how these interactions and the particle-to-particle distances might scale under confinement imposed by the "islands" of our interference-like patterns. Let us consider spherical paramagnetic particles of volume V_{p} that are free to move over a horizontal (xy) plane and are subject to an external magnetic field $\mathbf{B}(x, y)$ normal to this plane. Particle at position $\mathbf{r_i}$ will have an induced magnetic dipole moment $\mathbf{m_i} = \frac{\chi_{\mathrm{p}} V_{\mathrm{p}}}{\mu_0} \mathbf{B}(\mathbf{r_i})$, where χ_{p} is the effective magnetic susceptibility of an individual particle. These dipole moments are parallel, and therefore interparticle force is repulsive with magnitude:

$$F_{\mathrm{dd}}^{\mathrm{ij}}(r_{\mathrm{ij}}) = \frac{3\mu_0}{4\pi} m_{\mathrm{i}} m_{\mathrm{j}} \cdot \frac{2}{r_{\mathrm{ij}}^4} \qquad (1)$$

where μ_0 is is the magnetic permeability of free space, and r_{ij} is the distance between two particles. On the other hand, force acting on each dipole due to the gradient of the magnetic field will be pulling particles into the region of higher field:

$$\mathbf{F_m^i} = (\mathbf{m_i} \cdot \nabla)\mathbf{B}(\mathbf{r_i}) \qquad (2)$$

Let us assume that field is localized to some "island" to which the particles are attracted (as in our moiré -like patterns). To account for this confinement, we spatially rescale the field such that the overall magnetic flux through the (xy) remains constant. This can be done by "replacing" field $\mathbf{B}(\mathbf{r})$ with a new field $\widetilde{\mathbf{B}}(\mathbf{r}) = \left(\frac{1}{a^2}\right) \mathbf{B}\left(\frac{\mathbf{r}}{a}\right)$ where a is some constant. For the case when magnetic field distribution is a 2D Gaussian surface (see inset on Supplementary Figure 6), numerical simulation for 30 particles in this trap shows that scaling is $\tilde{r}_{\mathrm{ij}} \sim a^{1/3}$, as illustrated in

Supplementary Figure 6. Remembering this analysis is only a simple 2D approximation – that is, not capturing the intricacies of interactions between particles forming chains in 3D[3] and with details of the scaling changing with the shape of the trap –it indicates that as the confinement increases (e.g., the value of a becomes smaller), the particle-to-particle spacing is expected to decrease. As we will shortly see, this general result holds for much more accurate, 3D simulations.

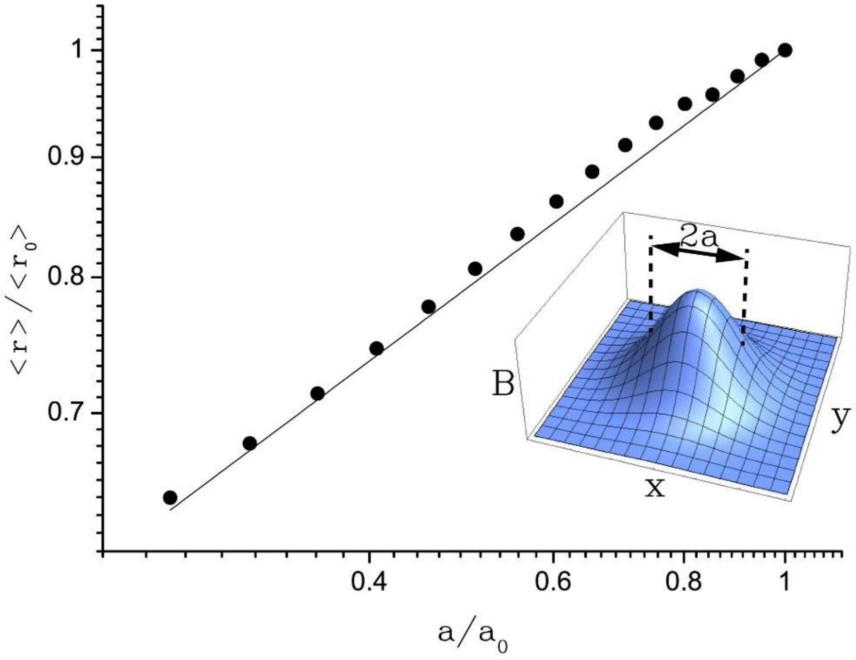

Supplementary Figure 6. Results of 2D simulations of interparticle distances between paramagnetic particles trapped in an "island" of high magnetic field. Parameter a measures effective geometric size a of the Gaussian-profile "island" (see inset). The average distance $\langle r \rangle$ from a given particle to its nearest neighbor is normalized by average distance $\langle r_0 \rangle$ between neighbors for some initial "island" size a_0. Notice the doubly-logarithmic scales. Filled circles are the results of numerical simulations; solid line is the function $\langle r \rangle / \langle r_0 \rangle = (a/a_0)^{1/3}$.

To study the dynamics of formation and structural details of patterns formed by chaining-up particles, we performed a series MD simulations in three dimensions. The results for both static and dynamic arrangements are summarized in Figures 5**,** Supplementary Figure 7 and Supplementary Movies 5, 6. As could be expected, the overall contours of the 3D patterns and the distribution of particles among different islands were similar to 2D models, but within each "island" the particles formed discrete, vertical columns/chains (cf. Figure 5, Supplementary Movies 5, 6) repelling each other via dipole-dipole interactions. The simulations reproduced the focusing effect in dynamic patterns, but also revealed some more subtle trends summarized in Supplementary Figure 7**:**

(i) More but also taller chains are formed when the number of particles increases (Supplementary Figure 7a).

(ii) The "quality" of the static patterns, q_s – defined as the ratio of particles attracted to the "islands" of field maxima, N_{is}, to the total number of particles in the system, N, and measured at a given time for a given strength of magnetic field – decreases as the viscosity of the medium increases. This decrease is faster than linear (Supplementary Figure 7b, t = 10 ms) and is due to the increasing drag forces experienced by the particles.

(iii) In "dynamic" patterns gradually rotated with respect to one another, the quality of "focused" structures decreases with increasing rotation rate (Supplementary Figure 7c). In this case, quality parameter, q_d, is defined as the number N_{isc} of particles attracted to the central "islands" (where these particles are "focused") divided by the total number of particles: $q_d = \frac{N_{isc}}{N}$,

and measured when the rotation angle between stamps reaches θ = 30° (see also Supplementary Movie 7).

We also observe that in the 3D simulations – similar to our "naïve" 2D analysis discussed above – separation between the particle chains slowly decreases with increasing confinement. This is seen, for instance, in Supplementary Movie 6 visualizing pattern evolution when the angle θ between two arrays of lines is steadily increased. In particular, increase from $\theta \sim 0^o$ to $\theta \sim 30^o$ (reducing the "long" axis of the island by ca. 2.5 times) causes the average separation between nearest-neighbor chains to decrease from about 3.8 µm to 2.3-2.4 µm (Supplementary Figure 8). With a scaling law derived from 2D simulations of a 2D Gaussian "island" (see Supplementary Figure 6), the comparable reduction of the island's size would correspond to a decrease to 2.8 µm – that is, quite close to what the full, 3D model predicts.

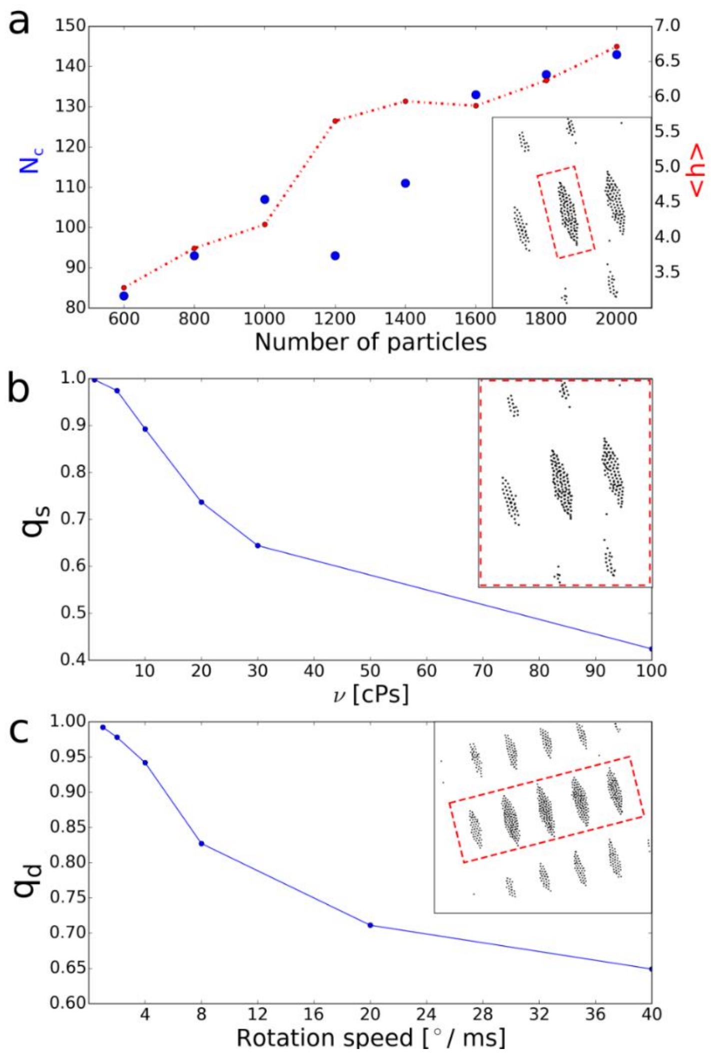

Supplementary Figure 7. Trends observed in 3D MD simulations. The simulations were performed for two stamps of parallel line features and **a,b** "statically" placed at θ = 30° on top of one another, and **c** "dynamically" rotated (in discrete steps of 2° per 2 ms) from θ = 0 ^{o} to 30°. Spacing between stamps was 10 µm but container height was limited to 9 µm. Number of particles in **b** and **c** was set to 1200 and 5000, respectively. Particles were initially distributed uniformly inside volumes of the following dimensions: **a, b,** 100 × 100 × 3.6 µm, **c,** 200 × 200 × 3,6 µm (width × length × depth). Properties of patterns that emerged in each case were quantified for regions of interest enclosed by the red dashed lines in the insets.

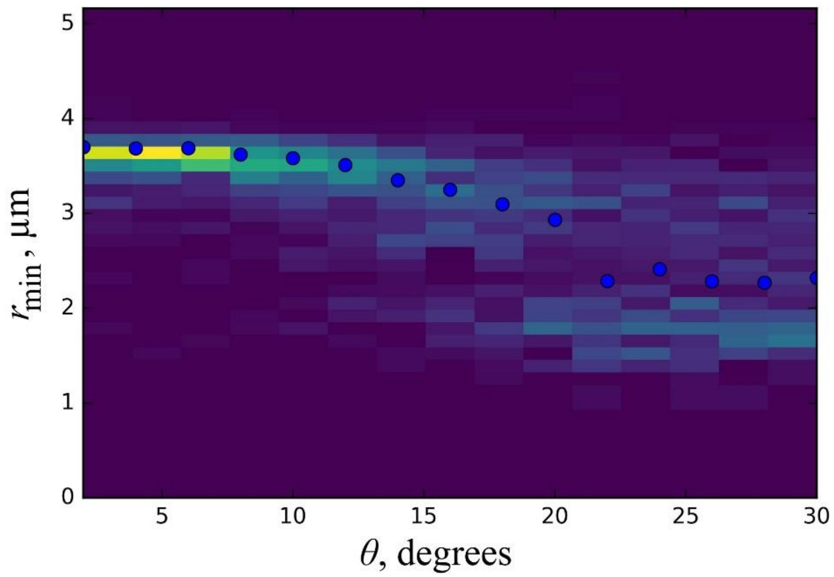

Supplementary Figure 8. Dependence of separation between nearest-neighbor particle chains on the angle θ between parallel line features. Color corresponds to the probability of observing a given chain separation at a given value of θ. Blue circles are median values. Data is obtained from 3D MD simulations in Supplementary Movie 6.

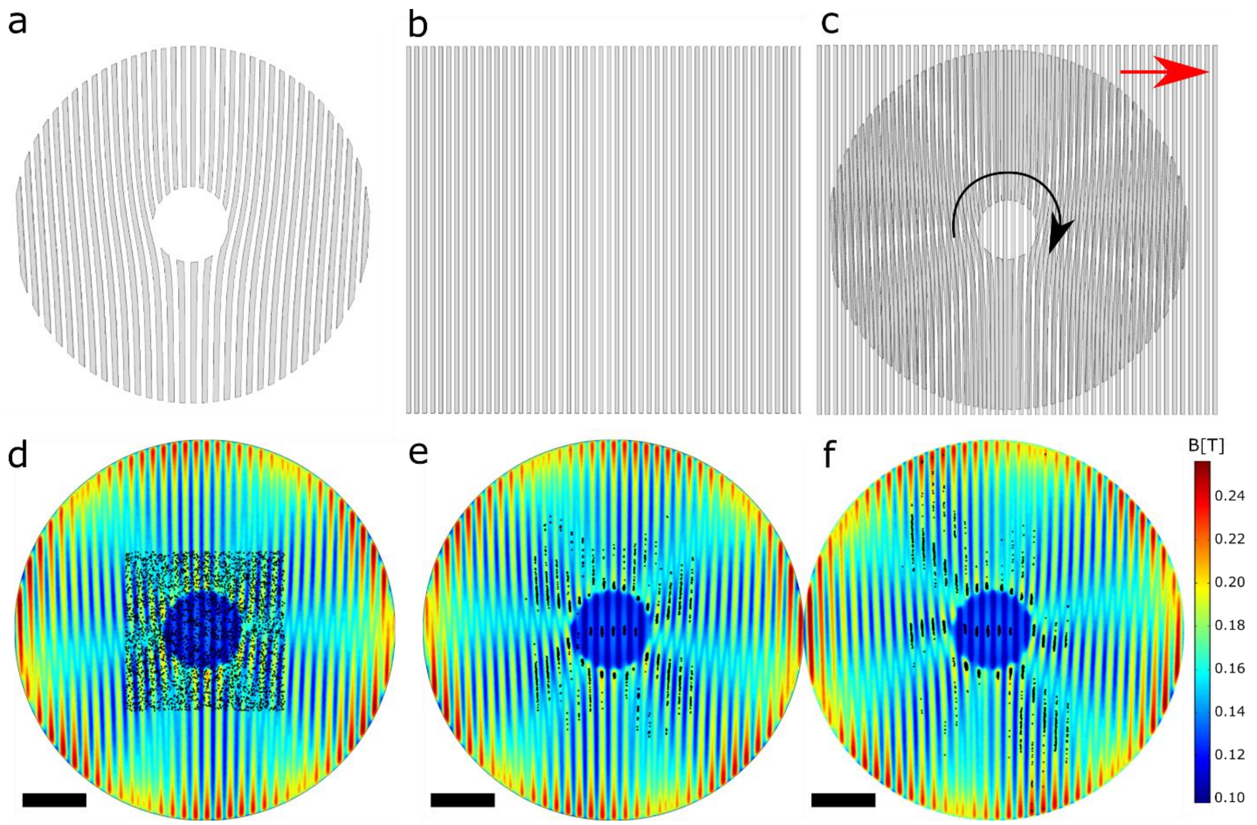

Supplementary Figure 9. Particle "rotor" via moiré-like magnetic patterns. Superposition of masks from **a,** and **b,** produces a pattern shown in **c** in which radially oriented variations (minima/maxima) in intensity are seen. When the lines are moved along the direction indicated by a black arrow, the radial pattern starts rotation, as indicated by the red arrow. Application of this concept to our system is demonstrated in MD simulations illustrated in **d-f**. The initial distribution (randomly over a square region) of particles at t = 0 is shown in **d**. Subsequent translation of the grid of lines at discrete steps of 0.5 µm per 2 ms causes the particles to orient into "blades" rotating clockwise. Shown here are two snapshots, at **e,** t = 100 ms and **f,** 250 ms. Please see Supplementary Movie 8 for full dynamics. The width of magnetic and non-magnetic features on both stamps was

~ 17 μm and the spacing between the stamps was 10 μm. All scale bars = 200 μm. Magnitude of magnetic field is indicated by the color scale.

Supplementary References:

1. Park, J. *et al.* Ultra-large-scale syntheses of monodisperse nanocrystals. *Nat Mater.* **3**, 891-895 (2004).
2. Gerber, R. Takayasu, M. & Friedlander, F. J. Generalization of HGMS theory: the capture of ultra-fine particles. *IEEE Trans. Magn.* **19**, 2115-2117 (1983).
3. Haghgooie, R. & Doyle, P. S. Transition from two-dimensional to three-dimensional behavior in the self-assembly of magnetorheological fluids confined in thin slits *Phys. Rev. E*, **75**, 061406 (2007)
