# Supplementary material for: Interference-like patterns of static magnetic fields imprinted into polymer/nanoparticle composites
Source: Nat Commun. 2017 Nov 16;8:1564. doi: 10.1038/s41467-017-01861-1 (PMC5691180; doi:10.1038/s41467-017-01861-1)
Supplement: Supplementary file 2 — Description of Additional Supplementary Information [file 41467_2017_1861_MOESM2_ESM.pdf]

Description of Additional Supplementary Files

File Name: Supplementary Movie 1
Description: **Rapid response of magnetic "supraparticles" to magnetic field**. The movie demonstrates that in the absence of magnetic field (00:00-00:05) particles perform Brownian motions. At 00:06 magnet (surface field ~0.4 T) is placed at the left-bottom corner causing particles to chain-up. When the magnet is moved, the chains follow the field. When magnet is removed (00:18) the chains disintegrate due to Brownian forces.

File Name: Supplementary Movie 2
Description: **Two-dimensional MD simulation of the organization of magnetic particles in a field modulated by two grids of magnetic lines inclined by $\theta = 30^o$.** Particles, initially uniformly distributed, move towards the closest rhombic regions of enhanced magnetic field. Ultimately, the particles redistribute almost evenly over all of high-field domains. The width of magnetic lines is 25 µm, number of particles is 4000, and the external field is 0.17 T.

File Name: Supplementary Movie 3
Description: **Two-dimensional MD simulation of magnetic particles in a magnetic field modulated dynamically by line-feature stamps rotated from 0° to 60°.** Initial distribution of particles is uniform. Shortly after simulation onset ($\theta = 0^o$), particles are attracted towards linear areas of enhanced magnetic field. As the rotation angle increases, the regions of field maxima "shrinks" into rhombi (e.g., at $\theta = 8^o$, 00:04) and the particle chains are "squeezed" into more compact structures. At $\theta > 8^o$, neighbouring lines from different patterns start to overlap and new rhomboidal field maxima appear – yet the particles, trapped at the initial rhombic regions, cannot "jump" to these new maxima because of potential barriers. Therefore, all particles are gradually being "focused" only over the original maxima. In the simulations, the stamp was rotated in discrete steps of 2° per 2 ms. The width of magnetic lines is 25 µm, the number of particles, 4000, and the external field is 0.17 T.

File Name: Supplementary Movie 4
Description: **Two-dimensional MD simulation of magnetic particles in a magnetic field modulated dynamically by stamps presenting patterns of concentric circles.** One of the stamps is translated horizontally (left to right) by 150 µm in discrete steps of 5 µm per 2 ms. The width of magnetic lines is 25 µm, number of particles is 4000, and field is 0.17 T. At the initial face-to-face configuration of stamps (00:00), uniformly distributed particles almost immediately organize over the superimposed circular field maxima. Akin to simulations in Supplementary Movie 3, gradual modification of the magnetic landscape leads to particle "focusing" in the shrinking local maxima with most particles in the maxima lying along the line perpendicular to the direction of patterns' motion.

File Name: Supplementary Movie 5
Description: **Three-dimensional MD simulation of pattern evolution in magnetic field modulated statically with line-feature stamps placed at $\theta = 30^o$.** Initially, 5000 particles are distributed inside a 200 µm × 200 µm × 3.6 µm box whereas total size of the simulation box is 500 µm × 500 µm × 9 µm (width × length × height). Compared to 2D simulations (cf. Supplementary Movie 2), particles organize into columns aligned perpendicularly to the surface of the stamps and repelling each other much stronger than individual particles. The width of magnetic and non-magnetic lines is 25 µm, and the imposed field is 0.17 T.

File Name: Supplementary Movie 6
Description: **Three-dimensional MD simulation of pattern evolution in magnetic field modulated dynamically with line-feature stamps rotated from 0° to 30°.** Initial distribution and the number of particles is the same as described in caption to Supplementary Movie 5. Particles organize within central "magnetic islands" as in 2D simulations. The difference with Supplementary Movie 5 is that the two stamps are being rotated while the magnetic particles organize into columns repelling by dipolar interactions. Stamp was rotated in discrete steps of 2° per 2 ms. The width of magnetic and non-magnetic lines is 25 µm, and the imposed field is 0.17 T.

File Name: Supplementary Movie 7
Description: **Two-dimensional MD simulation of pattern evolution at high rates of stamp's rotation.** In the initial part of the simulation ($\theta < 20^o$), the stamp is rotated in discrete steps of 2° per 2 ms. At the last three rotation steps (00:22-00:27), the angle is changed more rapidly, to 30°, 45° and 60° every 2 ms. The other simulation parameters are the same as in the system in Supplementary Movie 3. At large rotation rates, some of particles cannot "follow" the modified magnetic landscape, and are instead "trapped" in the neighbouring high-field domains. Simulation parameters are as in Supplementary Movie 6.

File Name: Supplementary Movie 8
Description: **Two-dimensional MD simulation of particles organizing into "rotor blades".** The superimposed patterns are those of circles/curves and lines shown in Supplementary Figure 8. The pattern of lines is translated horizontally from left to right in discrete steps of 0.5 µm per 2 ms. Scale bar is 200 µm. External field in the simulation is 0.17 T.
